# Trafficking of phagocytic peritoneal cells in hypoinsulinemic-hyperglycemic mice with systemic candidiasis

**DOI:** 10.1186/1471-2334-13-147

**Published:** 2013-03-25

**Authors:** Thais Fernanda de Campos Fraga-Silva, James Venturini, Maria Sueli Parreira de Arruda

**Affiliations:** 1 Faculdade de Ciências, Bauru, Departamento de Ciências Biológicas, Laboratório de Imunopatologia Experimental (LIPE), UNESP - Univ Estadual Paulista, Bauru, SP 17033-360, Brazil; 2 Instituto de Biociências, UNESP - Univ Estadual Paulista, Botucatu 18618-970, Brazil; 3 Faculdade de Medicina de Botucatu, UNESP - Univ Estadual Paulista, Botucatu, SP, Brazil

## Abstract

**Background:**

Candidemia is a severe fungal infection that primarily affects hospitalized and/or immunocompromised patients. Mononuclear phagocytes have been recognized as pivotal immune cells which act in the recognition of pathogens, phagocytosis, inflammation, polarization of adaptive immune response and tissue repair. Experimental studies have showed that the systemic candidiasis could be controlled by activated peritoneal macrophages. However, the mechanism to explain how these cells act in distant tissue during a systemic fungal infection is still to be elucidated. In the present study we investigate the *in vivo* trafficking of phagocytic peritoneal cells into infected organs in hypoinsulinemic-hyperglycemic (HH) mice with systemic candidiasis.

**Methods:**

The red fluorescent vital dye PKH-26 PCL was injected into the peritoneal cavity of Swiss mice 24 hours before the intravenous inoculation with *Candida albicans*. After 24 and 48 hours and 7 days of infection, samples of the spleen, liver, kidneys, brain and lungs were submitted to the microbiological evaluation as well as to phagocytic peritoneal cell trafficking analyses by fluorescence microscopy.

**Results:**

In the present study, PKH^+^ cells were observed in the peritoneum, kidney, spleen and liver samples from all groups. In infected mice, we also found PKH^+^ cells in the lung and brain. The HH condition did not affect this process.

**Conclusions:**

In the present study we have observed that peritoneal phagocytes migrate to tissues infected by *C. albicans* and the HH condition did not interfere in this process.

## Background

Systemic fungal infections in general, and of candidemia in particular, are severe infections that frequently affect immunocompromised patients including the patients with diabetes mellitus (DM) which correspond to 13%-21% of all candidemia episodes [1-3]. Experimental studies have shown that DM condition increases the susceptibility to candidiasis [4-6]; however, the immunological mechanisms involved in this process have not been extensively studied.

Deficiency in the host immune system is the major predisposing factor to *Candida* spp infection [7-9]. Macrophages are pivotal immune cells which act in the recognition of pathogens, phagocytosis, inflammation, polarization of adaptive immune response and tissue repair. They are deeply involved in the control and/or eradication of *Candida* infections [10,11].

Conchon-Costa et al. [12] demonstrated the possibility of controlling systemic candidiasis by activated peritoneal macrophages in the experimental model of Concanavalin A (ConA)-treated mice, using intraperitoneal route during systemic candidiasis. The researchers observed a decrease of the mortality of treated-mice challenged with a lethal dose of *Candida albicans*. This event was associated with increased TNF-α production, mannose receptor expression and candidacidal activity by peritoneal cells [12,13].

The participation of activated peritoneal cells in distant infections and/or inflammation has been suggested for some time. The migration of cells between the peritoneal cavity and inflamed [14-17], infected [16], neoplastic [18] and adipose tissues [19] have been demonstrated. Considering that the peritoneal cavity serves as an alternative site for immunotherapeutic procedures, the present study investigates the possibility that peritoneal phagocytes migrate to tissues infected by fungi. Because fungal infections are more frequent and difficult to remission in patients with DM, we also investigated the possibility that the hypoinsulinemic-hyperglycemic (HH) condition affects the peritoneal cell trafficking. According to our knowledge, this is the first study to explore migrating peritoneal phagocytes during systemic candidiasis.

## Methods

### Mice

Forty-five-day old male Swiss and C57Bl/6 mice from the Animal House of the Laboratório de Imunopatologia Experimental (LIPE), Departamento de Ciências Biológicas, Faculdade de Ciências, UNESP – Univ Estadual Paulista were used for the experiments. The animals received balanced diet and water *ad libitum*. All procedures were performed in accordance to the ethical standards established by the Brazilian College of Animal Experimentation (Colégio Brasileiro de Experimentação Animal - COBEA) and approved by the Committee for Ethics in Animal Experimentation of the Faculdade de Ciências – UNESP/Bauru.

### Fungi

*C. albicans* strain FCF 14 was originally obtained from the mycology collection of the Faculdade de Odontologia de Guaratinguetá, UNESP – Univ Estadual Paulista and was maintained in our mycological collections on Sabouraud dextrose agar (Difco Laboratories, Detroit, Michigan, USA) and monthly samplings in Sabouraud-dextrose medium.

### Experimental design

Sixty Swiss mice were distributed into 4 groups: Group Ca, which was composed of 18 *C. albicans*-infected mice; Group HH-Ca, composed of 18 HH-induced and *C. albicans*-infected mice; Group HH, composed of 18 HH-induced mice; and Control Group (CTL), composed of 6 free HH-induced and non-infected mice which were subjected to the same inoculation procedures using sterile saline solution (SSS).

### Induction of the HH condition

Alloxan administration in laboratory animals selectively destroys the insulin-producing pancreatic β-cells [20]. Mice were intravenously inoculated by alloxan (Sigma Chemical Co., St. Louis, MO, USA) in a single dose of 60 mg kg^-1^ of body weight into the caudal vein. Hyperglycemia was confirmed 48 hours (h) later using Accu-Check Advantage II blood glucose test strips (Roche, Mannheim, Germany). Only mice showing blood glucose levels 200 mg dl^-1^ were considered HH and included in the experiment. The glucose levels during the experiment were typically 400–600 mg dl^-1^ in HH mice. Control mice (Ca and CTL groups) were treated identically with a sterile saline solution and presented serum glucose levels ranging from 90 to 130 mg dl^-1^.

### Induction of systemic candidiasis

The *C. albicans* inoculum was obtained from a fungal suspension as previously described [21]. The fungal concentration was adjusted to 5.0 × 10^7^ viable *C. albicans* ml^-1^. The *C. albicans* suspension has been inoculated into the lateral tail vein. Mice of the Ca and HH-Ca groups were inoculated with 0.1 ml of this suspension and, in the HH-Ca groups the mice were inoculated two days after HH induction. Tests previously performed in our lab demonstrated that this inoculums size is not lethal in naïve Swiss mice and it reproduces a systemic fungal infection (data not shown).

### Determination of peritoneal cellular trafficking

#### Direct injection of PKH-26 PCL into the peritoneal cavity

Peritoneal phagocytes were labeled with the fluorescent vital dye PKH-26 PCL in accordance with the manufacturer’s protocol (Sigma). Briefly, 2.0 × 10^-6^ M PKH-26 PCL was diluted in the diluents B and 24 h before to the fungal inoculation, 0.1 ml of the PKH-26 PCL use solution was injected into the peritoneal cavity of each mouse.

#### Adoptive cell transfer assay

In order to confirm the peritoneal cells migration, we have used the adoptive cell transfer assay. As the Swiss mice are outbred strain, we have employed C57BL/6 inbred mice to perform the experiments as cell donor and recipient mice. A peritoneal lavage (PL) was performed in the donor mice, and the harvested cells were stained with PKH-26 PCL using the protocol according the previous item. After 30 minutes, the samples were centrifuged at 1500 rpm for 5 minutes and washed extensively with cold and sterile phosphate buffered saline (PBS). Naïve recipient mice were then intraperitoneally inoculated with 2 × 10^6^ PKH^+^ cells. After 24 h, these mice were intravenously challenged with 5 × 10^6^*C. albicans* and subsequently analyzed 24 h, 48 h and 7 days after the fungal inoculation.

#### Collection of biological samples

The mice were euthanized by carbon dioxide inhalation (CO_2_) 24 h, 48 h or 7 days after the fungal inoculation. PL was performed with cold and sterile PBS. To confirm the presence of PKH^+^ cells in the PL samples, smears were prepared with 0.1 ml of the sample. Spleen, brain, liver, lung and kidney samples were also collected from the euthanized mice.

#### Imprint

Using a scalpel, vertical sections were made on the tissues selected for the study. The pieces were pressed against glass slides and allowed to dry at room temperature. After washing them with PBS, the imprinted slides were mounted using Fluoroshield™ with DAPI (Sigma). The slides were analyzed with a fluorescence microscope (BX61, Olympus Optical, Tokyo, Japan). Following the identification of the migrating cells, the slides were subjected to confocal laser scanning microscopy analysis (TCS-SPE, Leica Microsystems, Mannheim, Germany), and images were captured using Leica LAS-AF Software.

### Determination of fungal load

The spleen, brain, liver, lung, kidney and PL samples were submitted to microbiological evaluation in order to determine the fungal load. After collection, the samples were weighed and macerated in 1.0 ml of sterile PBS. A volume of 0.1 ml was spread over culture plates containing Sabouraud-dextrose agar using a Drigalski T loop. The procedures were performed in duplicate. The plates were then sealed and incubated at 37°C for 3 days. The number of colony forming units (CFU) was normalized per gram of tissue.

### Statistical analyses

Statistical tests were performed using SigmaPlot Software version 12.0 for Windows (1995, Jandel Corporation, San Rafael, CA, USA), and the significance level established to verify the null hypothesis was 5.0%. The data passed in normality test (Shapiro-Wilk) and the comparison of two independent samples was analyzed using a *t* test [22].

## Results

### HH condition leads to an increase of fungal load

**Figure 1:**
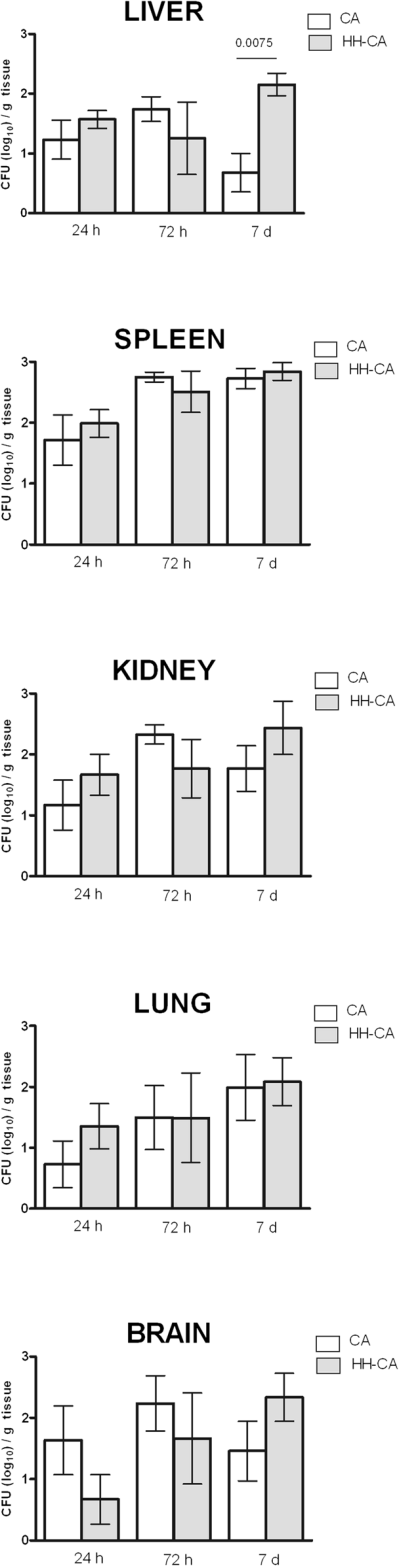
**Determination of fungal load in the Ca and HH-Ca groups.** The results are expressed as CFU (log_10_) per gram of tissue (*t* test; p < 0.05; n = 6/group and experimental moment).

The intravenous inoculation of *C. albicans* resulted in acute systemic dissemination affecting the kidney, spleen, liver, lung and brain. The HH condition did not affect the distribution of the fungi at 24 h and 48 h. However, on day 7, the mice of HH-Ca group exhibited higher fungal load in the liver than Ca group (Figure 1).

### Systemic infection by *C. albicans* enhances the spreading of labeled peritoneal phagocyte to infected tissue

Several methods have been used to determine cell trafficking such as adoptive cell transfer [14-16], *ex vivo* assays [19], cryogenic tissue evaluation [18,19] and flow cytometry [14,17,19,23]. In the preset study we used the PKH-26 PCL as tracing cell marker and imprinting technique to evaluate the migration of labeled cells. The vital fluorescent dye PKH-26 PCL provides strong fluorescence without causing functional damage to the cell. This dye induces the formation of aggregates, which allows the identification of phagocytic cells, such as macrophages and neutrophils. Several studies have used PKH-26 PCL for selective tracing of peritoneal phagocytes without staining phagocytes from other tissues or blood stream [24-27]. We previously performed some experiments to evaluate the PHK-26 PCL staining in frozen tissue sections and we observed more artifacts and lower fluorescence intensity than in fresh samples obtained from imprinting techniques (data not shown).

**Figure 2:**
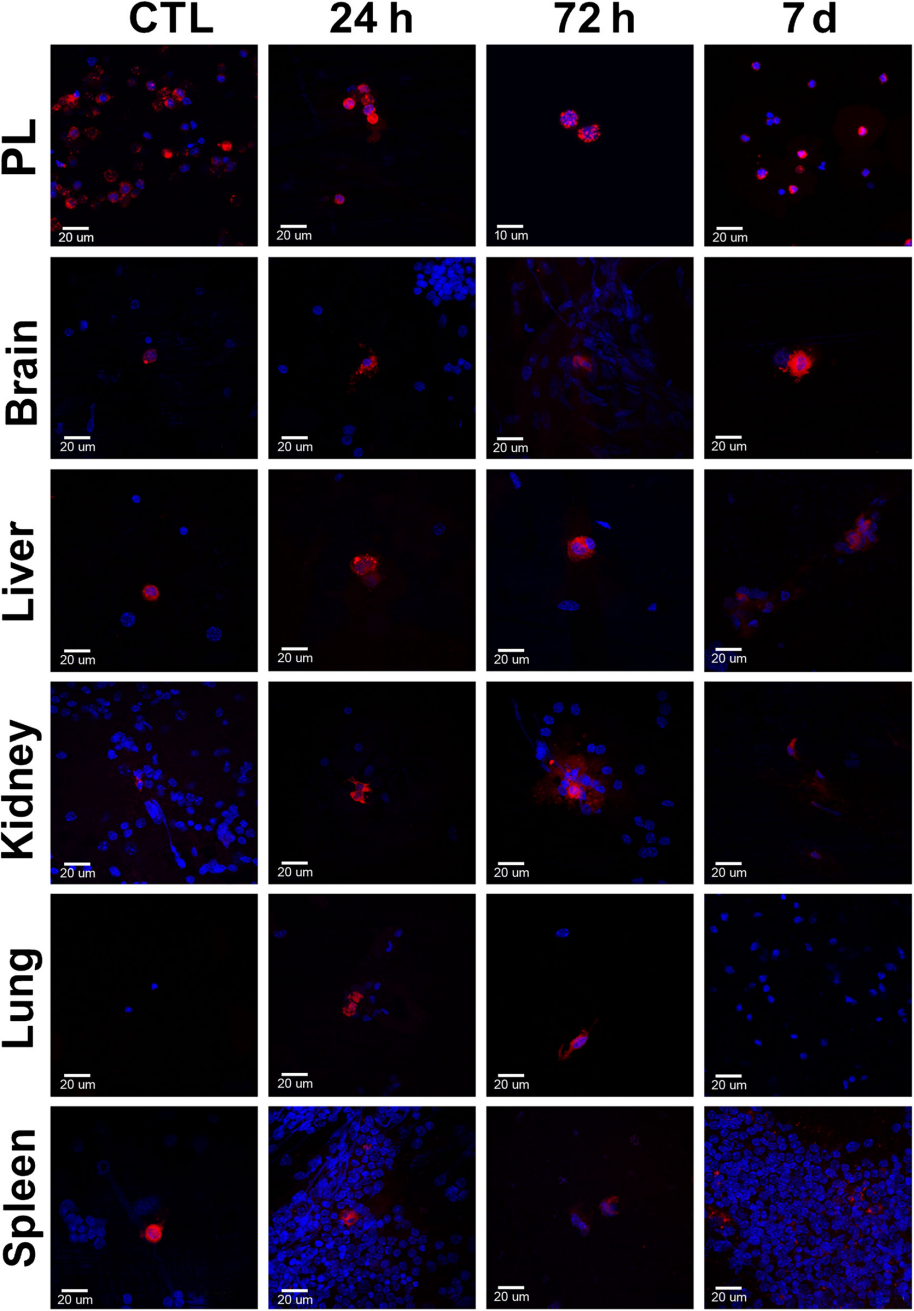
**Trafficking of peritoneal cells during systemic candidiasis.** Male Swiss mice were previously inoculated by intraperitoneal route with the fluorescent dye PKH-26 PCL and after 24 hours, the mice were intravenously challenged with 5 x 10^6^*C. albicans*. After 24 and 48 hours and 7 days, the mice were euthanized and samples of peritoneal cells, brain, liver, kidney, lung and spleen were subjected to fluorescence analysis. PKH-26 PCL: red; DAPI: blue.

In the present study, PKH^+^ cells were observed in the peritoneum, kidney, spleen and liver samples (Figure 2) from all groups, including the group of non-infected animals (HH and CTL groups). In the HH-Ca and Ca groups, PKH^+^ cells were also found both in the lung and brain. In the HH group, we observed no PKH+cells in the brain and in the CTL group, no PKH+cells in the lung. The cellular adoptive transfer assay confirmed the migration of PKH^+^ cells from the peritoneal cavity to all of the infected tissues at all of the time points analyzed in the present study (data not shown).

Furthermore, we observed morphological heterogeneity among migratory labeled cells (Figure 2).

## Discussion

The murine model for disseminated infection by *C. albicans* has been well characterized [28-30]. When fungal cells are intravenously inoculated by lateral tail vein, the fungus spreads rapidly, mimicking the infection that occurs when fungal cells enter the blood stream through the gastrointestinal tract, or an intravenous catheter. In our infected-mice, as previously described in non-progressive *Candida* hematogenous infection, the infection is controlled in the majority of the tissues, including the liver, spleen, heart and lungs but advances in the kidney and brain [30,31]. Here, we have amplified these findings using HH mice induced by alloxan. The choice of alloxan-induced HH mice has two rationales. The first one is due to DM represent an important import underlying disease in episodes of candidemia (13-21%) [1-3], and the second is because this condition interferes in the animal defense mechanisms, therefore making possible the study of the immunological alterations involved in the infections which undertake the patients with DM. In addition, the alloxan model, when used in the first hours after drug inoculation, mimics only the HH condition of DM and not the chronic disturbs; as a consequence, the data interpretation is specific to this abnormality. Our data has demonstrated an increase in the fungal load in the tissues of HH-mice (particularly the liver) 7 days after the inoculation. A larger fungal load has also been observed in the tissues of diabetic mice Mosci et al. [4], with an increased load which has been particularly prevalent in the liver of the HH-mice. The data reinforces the idea that the HH condition increases susceptibility to systemic candidiasis and suggests that this model could be an interesting option for investigating different aspects of this infection.

Several studies have shown that macrophages in a microenvironment rich in glucose exhibit diverse functional alterations, such as increased oxidative stress and enhanced transcription genes encoding for cytokines, growth factors and adhesive molecules [32]. These alterations have been triggered by increased formation of the advanced glycation end products (AGEs) [33]. Considering that the hyperglycemia affects the metabolism of these cells, we have come to the idea that other cellular alterations could be occurring which could affect the migration of these cells. We have identified an enhance in the spread of migratory peritoneal phagocytes during systemic candidiasis in naïve mice and that the HH condition did not interfere in this process. For this identification we have used the imprinting technique associated with PKH-26 PCL. The analysis of the peritoneal cell migration is not limited to these two methods. The PKH is an extensively used supply for cell tracking protocols [26,27,34], which could be used associated with other methodologies such as flow cytometry and two-photon excitation microscopy. The use of transgenic mouse line with fluorescent protein-labeled is another sophisticated methodology [35]. The imprinting is a simple methodology, routinely used for diagnostic in other situations, as in the evaluation of sentinel lymph nodes for lobular carcinoma of the breast cancer [36]. Several studies have demonstrated that the imprinting is superior to freezing, particularly in cases where the preservation of the tissue structure is not required [37-40]. Here, we have observed that imprinting provides a higher-quality analysis of a larger number of cells than the cryogenic sectioning and it is an interesting option for screening cell trafficking due to an easily and faster methodology.

## Conclusions

In summary, our results reinforce the migratory capacity of the peritoneal cells and demonstrate that the HH condition does not alter this process, enabling the use of the peritoneal cavity in immunotherapeutic approaches. Immunotherapy from this site has been considered a viable and promising alternative. This route has been used to immunize mice with *ex vivo*-treated dendritic cells for prostate cancer intervention [41]. Experimentally, ConA has been used to stimulate the peritoneal cell [12]; they have observed increased survival rate during lethal *C. albicans* hematogenous infection. Thus, even in patients with DM, the peritoneal cavity can be used for immunotherapeutic intervention, i.e., as a site for stimulating and macrophages to migrate from the peritoneum to the compromised tissues and to modulate the infectious process.

## Abbreviations

DM: Diabetes mellitus; HH: Hypoinsulinemic-hyperglycemic; COBEA: Brazilian college of animal experimentation; Group Ca: *C. albicans*-infected mice; HH-Ca: HH-induced and *C. albicans*-infected mice; Group HH: HH-induced mice; Control Group (CTL): Free HH-induced and non-infected mice; SSS: Sterile saline solution; PL: Peritoneal lavage; PBS: Phosphate buffered saline; CFU: Colony forming units.

## Competing interests

The authors declare that they have no competing interests.

## Authors’ contributions

TFCFS and JV performed the laboratory assays, participated in the sequence alignment, and drafted the manuscript. MSPA conceived the study, participated in its design and coordination, and helped to draft the manuscript. All authors read and approved the final manuscript.

## Pre-publication history

The pre-publication history for this paper can be accessed here:

http://www.biomedcentral.com/1471-2334/13/147/prepub
